# The influence of Antonovsky’s sense of coherence (SOC) and psychoeducational family intervention (PEFI) on schizophrenic outpatients’ perceived quality of life: a longitudinal field study

**DOI:** 10.1186/1471-244X-13-10

**Published:** 2013-01-07

**Authors:** Walter Gassmann, Oliver Christ, Jana Lampert, Hartmut Berger

**Affiliations:** 1 Vitos Philippshospital, Philippsanlage 101, Riedstadt, 64560, Germany; 2 Work and Engineering Psychology, TU Darmstadt, Alexanderstrasse 10, Darmstadt, 64287, Germany; 3 School of Applied Psychology, University of Applied Science Northwestern Switzerland, Riggenbachstrasse 16, Olten, 4600, Switzerland

**Keywords:** Multifamily intervention, Sense of coherence, Schizophrenia, Quality of life

## Abstract

**Background:**

Antonovsky’s sense of coherence (SOC) as well as psychoeducational interventions has a convincing impact on the quality of life (QOL) of patients suffering from schizophrenia. This study explores the influence of SOC on QOL among participants of a PEFI group (PG) compared to a control group (CG).

**Methods:**

In a quasi-experimental field study 46 schizophrenic outpatients had an option to participate together with their family members the PG (n = 25) or the CG (n = 21). They were assessed amongst others with the Quality of Life Questionnaire (WHOQOL-BREF), the Global Assessment of Functioning Scale (GAF), the Positive and Negative Syndrome Scale (PANSS) and the Sense of Coherence Scale (SOC-29). The efficacy of the PG on QOL was compared to the CG within two different SOC levels.

**Results:**

Before intervention patients with high SOC scores had significant higher levels in GAF and QOL and a trend of lower PANSS scores. The strongest relationship was found between SOC and QOL. Regarding the SOC level after intervention PG participants had higher QOL values than the CG within the last three measurements. The highest benefit due to QOL was observed within PG participants with high SOC scores.

**Conclusions:**

The results of the study suggest that SOC is a good predictive variable for clinical outcomes including QOL. Generally, the influence of the SOC level on QOL was stronger than the effect of PEFI. Hence schizophrenic patients with high SOC scores did benefit most from participating in a PG regarding their QOL. To optimize the effect of PEFI more efforts are needed to enhance the SOC of the participants. Altogether PEFI seems to be an important completion to the standard treatment for schizophrenic outpatients.

## Background

Schizophrenia is an extreme burden not only for the affected patients but also for their family members and partners [1]. The course of disease and the relapse rates are influenced as well by the frequently burdened familial climate as by the way of communication within the family members [2,3]. Particularly the feeling of being a burden for the family members can lead to a decrease of quality of life (QOL) within schizophrenic patients [4].

Patient-centred care for outpatients suffering from schizophrenia is still insufficient and does in many cases not comply with the wants and needs of the patients and their families. Treatment as usual for outpatients is almost limited to short contacts with a psychiatrist every four to six weeks and a more or less sufficient antipsychotic medication. International guidelines for schizophrenia additionally recommend family interventions or cognitive behavioral therapy. These interventions should be implemented beside an adequate antipsychotic medication during the post acute and remission state [5]. Sustainable treatment strategies focus not only on the reduction of symptoms and relapse rates but also on promoting a better QOL [6]. However in clinical practice there is a gap between the transfers from guidelines to implementation. Only 21% of the patients and only 2% of the relatives get the needed support in form of structured information about the disease, strategies of coping and crisis prevention [7].

These findings emphasize the necessity to design and implement new treatment strategies for patients with schizophrenic disorders. The efficacy of psychoeducational interventions has been confirmed in many cases [8,9] and especially multifamily intervention has shown to be a powerful instrument for mental health promotion [10].

Within the frame of our Health Promoting Hospital Standards for Mental Health Services [11] we designed a new psychoeducational family intervention (PEFI) for patients suffering from schizophrenia and their relatives [12]. PEFI imparts knowledge about the disease and adequate treatment options and beyond that improves the efficacy of individual and familial coping strategies. When these efforts are successful they may probably lead to a better QOL within the participating patients. But there is still an open question through which processes psychoeducational interventions aid in the management and treatment of schizophrenia.

Landsverk & Kane [13] proposed that one of the processes through which psychoeducation works is in maintaining and enhancing an individual’s sense of coherence (SOC). Until now only a few studies with different psychotherapeutic interventions approve this thesis [14,15]. The SOC is conceptualized by Antonovsky [16] and describes the adaptive capacity of a person as an individual view that recognizes the world as meaningful and predictable. The construct consists of the 3 components: comprehensibility, meaningfulness and manageability. This describes a person’s belief that internal and external stimuli in the course of life are comprehensive and predictable, resources are available to cope with the demands posed by these stimuli and these demands are meaningful challenges and worth of investment and engagement [17].

Latest studies showed that the SOC has a convincing impact on the QOL. A stronger SOC leads to a better QOL [18,19]. There are also recent findings that psychoeducational interventions can improve the QOL of schizophrenic patients [20-22]. Furthermore a previous study has shown that PEFI reduces the pre-post relapse rates within the participants and ameliorates the familial cohesion significantly. However the shortcoming of this study was the missing of a control group [12].

The present study is part of a pilot project for the implementation of an integrated health care unit for patients suffering from schizophrenia in the Vitos Philippshospital Riedstadt, Germany. The study wants to explore, whether the level of SOC works as a predictive variable for different clinical outcomes including QOL during a one-year period. Additionally the study wants to prove which patients dependent on their SOC level do benefit most from PEFI due to their QOL compared to a control group.

## Methods

In a quasi-experimental longitudinal field study 46 schizophrenic outpatients (see Table 1) had the option either to participate together with their family members the PEFI group (PG; n = 25) or the control group (CG; n = 21). Ethical approval to carry out the study was obtained from the Ethic Commission, Department of Psychology, Technische Universität Darmstadt (Darmstadt, Germany). All participants provided written consent.

**Table 1:** Socio demographic data of the sample

**Variables**	**PG (n = 25)**	**CG (n = 21)**	**p**
**Age**			0.082
in years M (SD)	34.2 (11.27)	40.2 (11.85)	
**Sex**			0.811
Male	14 (56.0%)	11 (52.4%)	
Female	11 (44.0%)	10 (47.6%)	
**Marital status**			0.293
Unmarried	17 (68.0%)	16 (76.2%)	
Married	8 (32.0%)	5 (23.8%)	
**Level of education**			0.261
Primary school	8 (32.0%)	8 (38.1%)	
Secondary school	7 (28.0%)	9 (42.9%)	
High School	10 (40.0%)	4 (19.0%)	
**Occupation**			0.364
Employed	11 (44.0%)	8 (38.1%)	
Day care center	4 (16.0%)	2 ( 9.5%)	
Housewife/-man	6 (24.0%)	9 (42.9%)	
Student	4 (16.0%)	2 ( 9.5%)	
**Living conditions**			0.513
Single	5 (20.0%)	7 (33.3%)	
Together with family	20 (80.0%)	14 (66.7%)	
**Diagnosis**			0.461
Schizophrenic	Psychosis (F 20)	17 (68.0%)	12 (57.1%)
Schizoaffective			
Psychosis (F 25)	8 (32.0%)	9 (42.9%)	
**Course of disease**			0.128
in years M (SD)	6.3 (7.58)	10.3 (9.39)	
Admission rate	.97 (.69)	.80 (.82)	0.451
(N of admissions per year)			

Abbr.: PG = Psychoeducational multi family group; CG = Control group; M = Mean; SD = Standard deviation.

All patients got treatment as usual in the psychiatric ambulance; additionally the PG received ten psychoeducational group sessions. Each PG was led by a psychiatrist and a psychologist and met once a week for two hours. Group sizes varied from 9 to 11 members (see Table 2). In the first five sessions information about the disease, possibilities of treatments and strategies of crisis prevention was given to the participants. In session 6 to 10 techniques from behavioral therapy like active listening, making legitimate demands, problem-solving and coping with stress were trained by role-playing to improve the communication within the families. After 6 months the participants were invited for booster-sessions to discuss the given information and the learned techniques.

**Table 2:** Number of PG participants

**PG (N)**	**Patients (N)**	**Relatives (N)**	**Group size (N)**
**1**	**4**	**7**	**11**
**2**	**3**	**6**	**9**
**3**	**5**	**6**	**11**
**4**	**4**	**5**	**9**
**5**	**4**	**7**	**11**
**6**	**5**	**6**	**11**

Abbr.: PG = Psychoeducational family group.

### Assessments

Socio demographic and disease related data were collected by a structured interview before intervention. Admission rates were conducted from the hospitals electronic basic documentation. Additionally all patients were assessed before intervention (T1), after three (T2), nine (T3) and twelve months (T4) with patient and clinician rated assessment scales.

### Patient rated scales

The Quality of Life Questionnaire (WHOQOL-BREF) has 26 items, each item scores from 1 = very bad to 5 = very good. Total score ranging from 26 to 130. It is a self-rated instrument and covers the dimensions physical well-being, psychological well-being, interpersonal relations and environmental well-being [23].

The Sense of Coherence Scale (SOC-29) has 29 items (each item scores from 1 = very often to 7 = rare or never; total score ranging from 29 to 201). It is a self-rated instrument and covers the dimensions comprehensibility, manageability and meaningfulness [24].

The Medication adherence rating scale (MARS) has 14 dichotome items. The scale is a self-rated instrument to assess medication adherence for psychiatric patients. This instrument covers as well the patients’ attitude towards medication as the actual medication-taking behavior [25].

The Clinical Global Impressions (CGI) rating scales are widely used as clinician rated instruments to assess symptom severity, treatment response and treatment efficacy in clinical studies and covers the dimensions severity of illness (CGI S), recovery (CGI R), effect of treatment (CGI E) and side effects (CGI SE). In this study these scales were transformed from a clinician rated form into a self rated form for the participating patients [26].

The Client Satisfaction Questionnaire (CSQ-8) has 8 items on a 4-point Likert scale. It is a self-rated instrument that wants to assess satisfaction with treatment and health care services [27].

### Clinician rated scales

The Global Assessment of Functioning Scale (GAF) is a clinician-rated instrument which scores from 1 = severe impairment of functioning to 100 = normal or unimpaired functioning [28].

The Positive and Negative Syndrome Scale (PANSS) has 30 items (each item scores from 1 = absent to 7 = severe; total score ranging from 30 to 210. The instrument is clinician rated and covers positive, negative and general psychopathological symptoms [29].

SPSS 15 was used for statistical analysis. Before intervention (T1) group comparisons between PG and CG and between patients with low and high SOC scores were done with independent samples t-tests. Subgroups of patients with low and high SOC levels were built by median splitting. Pearson correlations were used to prove the relations between all observed clinical variables. Additionally a multiple regression analysis was used to prove the best predictor variable regarding the QOL-outcome. After intervention (T2 – T4) an analysis of variance with repeated measurement was used to prove the effects of PEFI compared to the control group among patients with low and high SOC levels due to QOL.

## Results

### Baseline data

The socio demographic data of the sample showed no significant differences between PG and CG (see Table 1). But by means patients who choose for participation in the PG were rather younger, had a closer relationship to their family members or partners, and had rather a higher level of education as well as a shorter course of disease than patients who choose the CG.

High significant positive correlations were observable between QOL and GAF, CSQ and SOC and significant negative correlations between QOL and CGI E, CGI SE and PANSS. The strongest relationship between QOL and the observed clinical variables (r = .761; p = .000) was found between QOL and SOC (see Table 3). In addition the results of a multiple regression analysis showed the highest beta score (β = .520; T = 4.585; p = .000) between SOC and QOL within all examined variables. As well between QOL and CGI E, CGI SE and CSQ significant but lower p-values were observable (see Table 4).

**Table 3:** Pearson correlations at Baseline (T1)

	**GAF**	**PANSS**	**SOC**	**MARS**	**QOL**	**CGI S**	**CGI R**	**CGI E**	**CGI SE**	**CSQ**
**GAF**		-.749**	.544**	**.076**	.483**	**-.135**	**-.076**	-.452**	**-.328**	**.305**
**PANSS**			-.505**	**-.030**	-.374*	**.195**	**.155**	.507**	**.315**	-.427**
**SOC**				**.154**	.762**	**-.196**	**-.283**	-.472**	**-.329**	.344*
**MARS**					.310*	**.163**	**-.075**	**-.201**	-.413*	.513**
**QOL**						**-.142**	**-.221**	-.676**	-.378*	.616**
**CGI S**							.510**	**.188**	**.237**	**-.028**
**CGI R**								**.307**	.383*	**-.150**
**CGI E**									.397*	-.779**
**CGI SE**										-.455**

* p < 0.05; ** p < 0.01.

Abbr.: GAF = Global Assessment of Functioning; PANSS = Positive and Negative Syndrome Scale; SOC = Sense of Coherence Scale; MARS = Medication Adherence Rating Scale; QOL = Quality of Life Scale; CGI S = Clinical Global Impressions – Severity; CGI R = Clinical Global Impressions – Recovery; CGI E = Clinical Global Impressions – Effect of Treatment; CGI SE = Clinical Global Impressions – Side effects; CSQ = Client Satisfaction Questionnaire.

**Table 4:** Multiple regression analysis on QOL at Baseline

**Variable**	**Beta score**	**T**	**Variable**
COD	-.038	-.345	.734
GAF	-.120	-.754	.459
PANSS	-.119	-.776	.446
SOC	.520	4.585	.000**
MARS	.194	1.497	.149
CGI S	-.188	−1.552	.135
CGI R	.025	.204	.840
CGI E	-.294	−2.406	.025*
CGI SE	.258	2.139	044*
CSQ	.317	2.541	.019*
AR	.058	.489	.630

* p < 0.05; ** p < 0.01.

Abbr.: QOL = Quality of Life; COD = Course of Disease; GAF = Global Assessment of Functioning; PANSS = Positive and Negative Syndrome Scale; SOC = Sense of Coherence; MARS = Medication Adherence Rating Scale; CGI S = Clinical Global Impressions – Severity; CGI R = Clinical global Impressions – Recovery; CGI E = Clinical Global Impressions – Effect of Treatment; CGI SE = Clinical Global Impressions – Side Effects; CSQ = Client Satisfaction Questionnaire; AR = Admission Rate.

In this study, a low SOC level includes SOC scores from 125 points and lower, in contrast a high SOC level includes SOC scores higher than 125 points. A group comparison of patients with high and low SOC scores showed that patients with high SOC scores had a significant higher level in GAF, QOL and CGI E than patients with low SOC scores, a significant lower admission rate and also a trend of lower PANSS scores (see Table 5).

**Table 5:** Group comparison of patients with low and high SOC levels

**Variable**	**SOC l/h**	**N**	**Mean**	**SD**	**T**	**p**
**Course of disease**	**low**	**23**	**7.09**	**7.56**	**-.820**	**.417**
	**high**	**23**	**9.17**	**9.58**		
**Admission rate**	**low**	**23**	**1.15**	**.91**	**2.501**	.016 *
	**high**	**23**	**.63**	**.43**		
**GAF**	**low**	**23**	**46.17**	**9.92**	**−3.417**	.001**
	**high**	**23**	**57.78**	**12.92**		
**PANSS**	**low**	**22**	**86.41**	**16.49**	**1.296**	**.200**
	**high**	**23**	**79.04**	**21.22**		
**SOC**	**low**	**23**	**102.13**	**20.48**	**−8.797**	.000 **
	**high**	**23**	**147.74**	**14.09**		
**MARS**	**low**	**23**	**8.35**	**3.12**	**-.807**	**.424**
	**high**	**23**	**9.04**	**2.70**		
**QOL**	**low**	**23**	**51.75**	**12.00**	**−5.037**	.000 **
	**high**	**23**	**69.40**	**11.75**		
**CGI S**	**low**	**23**	**3.26**	**2.20**	**.469**	**.261**
	**high**	**21**	**3.00**	**1.34**		
**CGI R**	**low**	**21**	**2.90**	**1.48**	**1.608**	**.723**
	**high**	**22**	**2.18**	**1.46**		
**CGI E**	**low**	**18**	**1.94**	**.53**	**3.346**	.002 *
	**high**	**20**	**1.25**	**.71**		
**CGI SE**	**low**	**17**	**2.53**	**.80**	**1.463**	**.152**
	**high**	**23**	**2.13**	**.92**		
**CSQ**	**low**	**23**	**22.39**	**4.03**	**-.987**	**.329**
	**high**	**23**	**24.04**	**6.94**		

* p < 0.05; ** p < 0.01.

Abbr.: GAF = Global Assessment of Functioning; PANSS = Positive and Negative Syndrome Scale; SOC = Sense of Coherence Scale; MARS = Medication Adherence Rating Scale; QOL = Quality of Life Scale; CGI S = Clinical Global Impressions – Severity; CGI R = Clinical Global Impressions – Recovery; CGI E = Clinical Global Impressions – Effect of Treatment; CGI SE = Clinical Global Impressions – Side effects; CSQ = Client Satisfaction Questionnaire.

### Evaluation of QOL values

**Figure 1:**
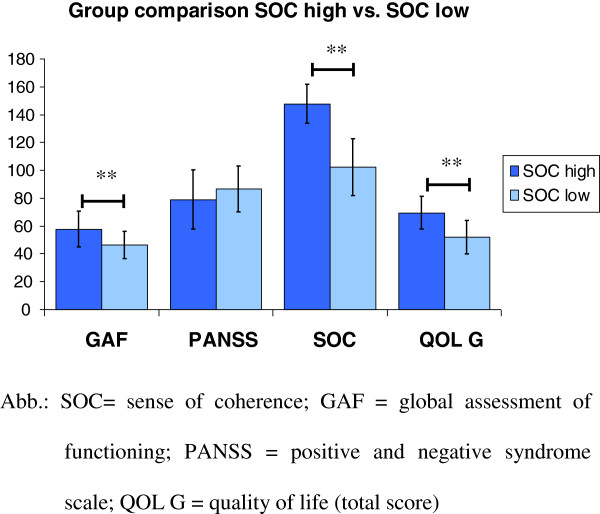
Effect of PG versus CG on QOL of schizophrenic patient with low and high SOC levels.

Before intervention (T1) the quality of life scores over all subgroups showed a mean value of M = 60.58 (SD = 14.75). At baseline independent samples t-tests showed no significant differences between PG and CG in the low and high SOC subgroups. As well PG participants with high as with low SOC scores showed higher QOL values compared to the CG within the last three measurement points (see Figure 1). The highest over all progress in the last three measures was observed within PG participants with high SOC scores. An analysis of variance with repeated measurement showed a significant effect of test intervals (F_3,34_ = 4.214; p = .012; effect size η2 = .271), a marginal significant interaction of test intervals and intervention (F_3_ = 2.189; p = .093; effect size η2 = .057) and a significant main effect of SOC levels (F_1_ = 10.064; p = .003; effect size η2 = .218).

## Discussion

The results of this study confirm previous studies regarding SOC, QOL and multifamily intervention [10,12,19,22]. At baseline, within all observed variables the strongest relationship was found between SOC and QOL. Additional findings of a significant influence of CGI E, CGI SE and CSQ on QOL had not been considered in this study, but should be respected in other therapeutically contexts.

As expected patients with high SOC scores had a higher level in QOL [18]. Furthermore patients with high SOC scores had a lesser admission rate, a higher level of general functioning, fewer psychopathological symptoms and a stronger perceived effect of their medical treatment. All these findings confirm the hypothesis that the SOC is a good predictor for clinical outcomes and especially for QOL.

Before intervention all participants of the study showed lesser QOL scores compared to the German norm population [22], but by means patients with higher SOC scores had higher QOL values than patients with lower SOC scores. PG participants with high SOC scores could enhance their QOL scores within a one year period to the same level as the German norm population. In contrast, PG participants with low SOC scores had also a remarkable enhancement due to their QOL within a one-year period, yet they did not reach the same level of QOL scores than the PG participants with high SOC scores.

However the results of an analysis of variance showed that the effect size of PEFI is much lesser than the effect size of SOC. To optimize the effect of this intervention more efforts are needed to enhance the SOC of the PG participants, especially of those with low SOC scores. With this enhancement the possibility of reducing the standard deviation might be probable.

Altogether PEFI seems to contribute to an enhancement of QOL beside the standard treatment for patients suffering from schizophrenia. Therefore this intervention should be disposable in outpatient care units for schizophrenic patients. Psychoeducational family interventions have merely an indication for a selected sample of patients, especially for those patients who have both a close relationship to their families and yet less experience and knowledge about the disease and the treatment options. Likewise the transferability of the findings in other contexts may be reduced because of the rather small sample size and the fact that the participating patients were comparatively less cognitive impaired. Additionally, in fact that all patients were recruited proximately before discharge most of the patients were still in a post acute state and had temporary fluctuations in symptoms and their course of recovery which may have an effect to the stability of patients ratings within the used self-reported clinical scales.

## Conclusion

To conclude this study wants to investigate the real terms in every day clinical practice. So of course there may be a stronger external or ecological than internal validity. Randomization or a waiting control group might have been enhanced the internal validity and therefore also the effects of PEFI but organizational limitations have prevented these approaches.

## Competing interests

No competing interests occurred.

## Authors’ contributions

All authors read and met the ICMJE criteria for authorship and agree with the results and conclusions. WG designed the study; OC and WG analyzed the data; WG, HB and JL collected data; WG and OC wrote the first draft of the paper; JL and HB contributed to the writing of the paper; WG and OC contributed to analysis and interpretation of the data; WG, HB, JL and OC contributed to the discussions on the design and interpretation of the study. All authors read and approved the final manuscript.

## Pre-publication history

The pre-publication history for this paper can be accessed here:

http://www.biomedcentral.com/1471-244X/13/10/prepub
